# Factors Influencing Therapeutic Observance in Diabetic Subjects in the Province of Essaouira (Morocco): A Cross-Sectional Study

**DOI:** 10.4314/ejhs.v32i4.11

**Published:** 2022-07

**Authors:** Khaoula Houguig, Samia Rkha, Mahassine Rayadi, Nadia Ouzennou

**Affiliations:** 1 Pharmacology, Neurobiology, Anthropobiology and Environment Laboratory, Department of Biology, Faculty of Sciences Semlalia, Cadi Ayyad University, Marrakech, Morocco; 2 ISPITS, Higher Institute of Nursing and Technical Health, Marrakech, Morocco; 3 Endocrinology service, Sidi Mohamed Ben Abdellah hospital, Essaouira, Morocco

**Keywords:** Diabetes, medical treatment, observance, Morocco

## Abstract

**Background:**

Therapeutic observance remains a major problem in managing diabetic subjects, just like in other pathologies treated by medication and lifestyle modification. This study aims to determine the rate of therapeutic observance among diabetic subjects in the province of Essaouira and to identify the factors that influence it.

**Methods:**

A cross-sectional questionnaire survey was conducted among 498 type 1 and 2 diabetic subjects, regularly being checked at different health centers in the province of Essaouira (Morocco).

**Results:**

Almost a quarter of the surveyed subjects (23.3%) had poor observance. The results of the binary logistic regression model show that, in order of importance, observance is associated with six factors: Availability and access to medical treatment (_Odds Ratio_ OR: 3; 95% CI confidence interval [1.78–5.03]); the side effects related to the treatment (OR: 2.60; 95% CI [1.65–4.09]); the family support (OR: 1.58 ; 95% CI [0.95–2.61]); duration of diabetes (OR: 0.55 ; 95% CI [0.34–0.88]); the age (OR: 0.50 ; 95% CI [0.30–0.82]); awareness level about the disease (OR: 0.43 ; 95% CI [0.21–0.90])

**Conclusion:**

The results of the present study have allowed us to identify several factors that can influence therapeutic observance, that prove necessary to be considered and acted upon.

## Introduction

No country on the globe is free from some burden and predicaments linked to diabetes (1). In Morocco, for instance, diabetes is the leading cause of blindness, the leading cause of end-stage renal failure, the leading cause of lower limb amputations (2).

The UK Prospective Diabetes Study shows that a 1% reduction in glycated haemoglobin is associated with a 30% decrease in the relative risk of developing complications, an 18% drop in the risk of heart attacks and a 25% decrease in the rate of diabetes-related mortality (3,4).

To control, minimize and reduce the costs of diabetes complications, effective and rigorous management based on good therapeutic observance and lifestyle habits should be considered as soon as the patient is diagnosed positive (5).

Therapeutic observance can be defined as the degree of adequacy between the subject's behavior and his physician's recommendations (6). Medical prescriptions related to diabetes are generally described as complex and burdensome (e.g., multiple injections of insulin, adaptation of insulin doses to different circumstances) (5).

To improve glycaemic control, these prescriptions must be strictly and rigorously followed, which involves important changes in the diabetic subject's lifestyle. However, several studies at the international level (7,8, 9), as well as on the national scheme (10,11,12) show that diabetic subjects do not follow the medical treatment rigorously and that many factors constrain the process of following the medical prescriptions (13).

These factors may be related to the socioeconomic and cultural characteristics of the diabetic subjects (14,15,16), the particularities of the disease and treatment (6,12,17), the attitudes of the physician and the health care facilities (18,13,11).

In Morocco, no study has evaluated these factors in a socioeconomically disadvantaged province, so it would only be relevant to determine the rate of therapeutic observance among diabetic subjects in the province of Essaouira and to identify the factors intervening with it.

## Subjects and Methods

This cross-sectional study comprises 12 health centers in Essaouira province, between January and December 2018. This city presents one of the most vulnerable provinces in the Marrakech-Safi region (19). Diabetic subjects were included in the study based on an accidental probabilistic sampling. A face-to-face interview was conducted with 498 diabetic subjects that are being managed at these centers. They expressed their favorable opinion to take a part of this study. The data collected concerns socio-demographic, psychosocial, and pathology-related characteristics of the diabetic subjects, based on a structured and detailed questionnaire. The questionnaire was developed, tested, and validated by the research team and in accordance with the literature review. With reference to both the Moroccan nutritional guide (20) and the thematic guide “Therapeutic education of type 2 diabetics” (21), we present in table 1 a summary of the therapeutic observance conditions along with an interpretation for the findings. For statistical testing, we considered diabetic subjects with high observance as “observant” and those with medium and low observance as “non-observant”. Observance in terms of hygiene and dietary rules was also evaluated (22).

**Table 1:** Conditions for evaluation of therapeutic observance

Questions to assess medication adherence	Answer	Interpretation of results
**1.** During the past four weeks, have you forgotten to take your medication?	Yes or No	Each “No” answer was counted as “0” points and “Yes” as “1” point.
**2.** During the past four weeks, have you ever delayed taking your medication?	Yes or No	“**high observance**” if a total score is equal to “0”.
**3.** During the past four weeks, Have you ever stopped your medication because you feel better?	Yes or No	“**medium observance**” if a total score is equal to “1 or 2”.
**4.** During the past four weeks, have You ever not taken your treatment because, you feel like your treatment is doing you more harm than good?	Yes or No	“**low observance**” if a total score is equal to “3 or 4”.

**Variable Definitions**:

**Professional activity**: Diabetes subjects' Professional activity is defined based on four socio-professional categories (SPCs):

SPC1: no occupation; SPC2: artisans, employees, workers, shop assistants, farmers, employed workers, laborers, drivers...; SPC3: officials, middle-level professionals...; SPC 4: liberal professions, higher management, major merchants... (23)

**Monthly family income**: Monthly family income refers to the sum of the monthly wages and returns acquired by all household members at the time of the interview and is expressed in MAD (is the official monetary currency of Morocco ) (24).

**Awareness level about the disease**: The knowledge and information the diabetic subject has on the disease was evaluated. Accordingly, a subject is considered “informed” when s/he knows some of diabetes' potential complications and that s/he acknowledges that by not following therapy, the risk of these complications occurring is greater. Besides, s/he is also aware of the importance of hygiene and dietary rules.

**Family support**: The involvement and commitment of the family to support the diabetic patient to better manage his condition were evaluated by asking the following question: Are you satisfied with the support you receive from your close ones (spouse, partner, family, friends...)?

**Availability and access to medical treatment**: The diabetic subject is considered to have problems with access to medical treatment when s/he cannot find the appropriate treatment at the convenient dispensary, under claims that there is a stock shortage, or when s/he declares that s/he has no means to buy it from the drugstore.

**Inclusion criteria**: The interviewees were diagnosed with type 1 or 2 diabetics, and they follow treatment procedures in the Essaouira province health centers, whether or not they complied with the treatment recommendations and agreed to participate in the study.

**Exclusion criteria**: Diabetic subjects under 18 years of age, pregnant women with diabetes, and subjects who were not under a treatment period, have been excluded from the study.

**Statistical analysis**: Data from this survey are typed into IBM® SPSS® 18 (Statistical Package for the Social Sciences) software for processing. Frequencies, means, and standard deviations are calculated and a chi-square test is performed to determine associations between categorical variables. Besides, binary logistic regression is performed to capture the weight of variables associated with medication observance. In fact, variable adjustment is required for the logistic regression model. As far as age is concerned, this survey considers “less than or equal to 60 years” versus “greater than 60 years”. Observations containing at least one missing data item have been removed. Statistical significance is set at the 5% level. Meanwhile, Excel 2007 is used as a means to put the figures together.

**Ethics approval and consent to participate**: The survey is conducted in full respect of local ethical considerations, namely obtaining authorization [N°8874] from the regional and local services of the Moroccan Ministry of Health. The ethics committee (Moroccan Association for Research and Ethics) granted the project approval [IRB00012973]. After informing the diabetic subjects of the purpose of this research, they clearly and willingly expressed their consent and agreement to participate in the study. All participants gave their informed consent and all data is thus collected anonymously.

## Results

**Figure 1:**
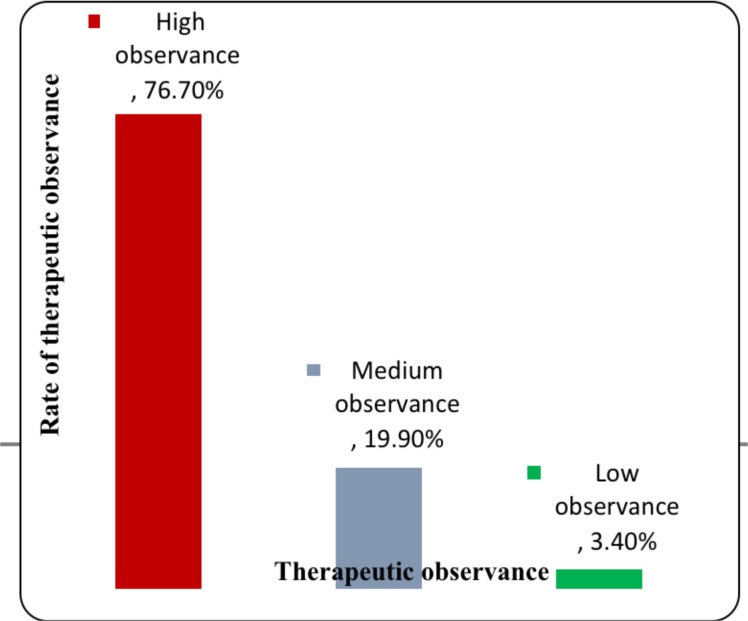
The prevalence rate of therapeutic observance

The study comprises a total of 489 diabetic subjects. Table 2 presents the survey subjects' socio-demographic characteristics. Women are slightly overrepresented (78.1%) and almost half of the respondents belong to the age group between 40–60 years. Most subjects have never attended school (82.2% of women and 17.8% of men) [p<0.001]. A quarter of the respondents are active employees and 87% receive a monthly income of less than 2,000 MAD per family. The duration of diabetes is mainly 8.6 years (SD = 7 years). Mean glycated hemoglobin is greater than 7% in 62.7% of diabetics. Hypertension and dyslipidemia were the most common diabetes comorbidities associated (25.9% and 11% respectively). Furthermore, with regards to the complications, 10.6% had retinopathy, 3.4% had neuropathy and 2% had nephropathy. Therapeutic observance is considered “high” when the total score equals “0”, therapeutic observance is “medium” when the total score equals “1 or 2”, and therapeutic observance is “low” when the total score equals “3 or 4”. The prevalence rate of therapeutic observance is eventually summarized in figure 1.

**Table 2:** Description of socio-demographic characteristics of the study population (n=498)

	Population (n=498)	
	
Characteristics	Effective	%
**Gender**		
Male	109	29.1
Female	389	78.1
**Age groups (years)**		
<40	39	7.8
[40–60]	281	56.4
>60	178	35.7
**Instruction level**		
Illiterate	415	83.3
Primary school	46	9.2
High school and university	37	7.4
**Professional activity**		
SPC1	374	75.1
SPC2	111	22.3
SPC3	11	2.2
SPC4	2	0.4
**Monthly family income**		
<2 000 MAD	437	87.8
[2 000–4 000 MAD]	43	8.6
>4 000 MAD	18	3.6
**Marital Status**		
Married	356	71.5
Divorced	28	5.6
Widowed	97	19.5
Single	17	3.4
**Place of residence**		
Urban	304	61
Rural	194	39

**SPC**: Socio-professional categories; **MAD**: official monetary currency of Morocco

Regarding psychosocial characteristics, only 17.1% of the interviewees were well informed about the disease. More than three-quarters (77.3%) of the respondents are supported by their entourage and 57.6% have a good observance of hygiene and dietary rules. In addition, 20.5% have problems with access to medical treatment.

To identify the factors influencing therapeutic observance, we resort to the following variables: gender, age, monthly family income, duration of diabetes, side effects related to the treatment, to be informed about the disease, family support, observance of hygiene and dietary rules, problems of access to medical treatment. In the univariate analysis, it was noted that subjects who reported side effects of treatment as well as those who had problems with access to medical treatment were the least observant [p<0.001]. A statistically significant difference was also recorded for “awareness level about the disease” and “age” [p<0.01], “family support” and “duration of diabetes” [p<0.05] (Table 3). In binary analysis, subjects who reported problems with access to medical treatment were 3 times more susceptible to not adhering correctly to their medical treatment. Whereas, subjects with treatment side effects were more than 2 times more susceptible to being non-observant. On the other hand, the subjects who declared that they are supported by their entourage were almost twice as likely to be observant (Table 4).

**Table 3:** Association of therapeutic observance with different variables (n=498)

Variables	Therapeutic observance N=498		p-value*
		
	Yes n=382	No n=116	
**Gender**			
Female	293(75.3)	96(24.7)	0,16
Male	89(81.7)	20(18.3)	
**Age groups (years)**			
<40	24(61.5)	15(38.5)	0,003
[40–60]	208(74)	73(26)	
>60	150(84.3)	28(15.7)	
**Monthly family income**			
<2000Dh	340(77.8)	97(22.2)	0,27
[2000–4000Dh]	29(67.4)	14(32.6)	
>4000Dh	13(72.2)	5(27.8)	
**Duration of diabetes**			0,047
≤8–6 years	214(73.5)	77(26.5)	
>8–6 years	168(81.2)	39(18.8)	
**Side effects related to the treatment**			
Yes	125 (66.1)	64(33.9)	
No	257(83.2 )	52(16.8)	0,000
**Observance of hygiene and dietary rules**			
Good	226(78.7)	61(21.3)	
Poor	156(73.9)	55(26.1)	0,2
**Awareness level about the diseases**			
Good	75(88.2)	10(11.8)	
Poor	307(74,3)	106(25,7)	0,006
**Family support**			
Good	305(79.2)	80(20.8)	
Poor	77(68,1)	36(31,9)	0,014
**Problem of access to medical treatment**			
Yes	65(63.7)	37(36.3)	0,001
No	317(80,1)	79(19,9)	

**Table 4:** Bivariate analysis of factors influencing therapeutic observance

Variables	Odds ratio*	Confidence Interval 95%	*p*-value
**Age**	0.502	0.304–0.829	0.007
**Duration of diabetes**	0.554	0.347 – 0.886	0.014
**Side effects related to the treatment**	2.607	1.658 – 4.097	0.000
**Awareness level about the disease**	0.438	0.213 – 0.902	0.025
**Family support**	1.582	0.959 – 2.610	0.072
**Problem of access to medical treatment**	3.000	1.789 – 5.031	0.000

* Odds ratio calculated using logistic regression. A value of p < 0.05 is considered significant

## Discussion

In the present study, observance prevalence reached 76.7%. Other epidemiological studies have reported a rate ranging from 47% to 84% (14,25,7). The age of diabetic subjects is a determining factor in non-observance. Subjects under 60 years of age are more susceptible to being non-observant. In addition, the observance quality is better in subjects whose condition has been diagnosed for more than 8.6 years. Indeed, when the diagnosis of the disease is recent, the diabetic subject is not aware of the risk of developing complications and that these complications may become more complex with age, the subject may believe that he does not need treatment or that this latter is quite ineffective (25). Similar results from other studies have indicated that younger age correlates with nonobservance (27,28), others have correlated it with older age (16). In the present study, we did not find a significant difference between both genders, or relating to monthly family income.

Side effects, such as digestive discomfort and hypoglycemic episodes, recorded in 16% and 15% of cases respectively, they are thus considered major determinants of nonobservance. Diabetic subjects who reported that their treatment was binding prove more than twice as susceptible to being non-observant (OR=2.6 ; 95% CI [1.65–4.09]). Following the appearance of side effects, the diabetic subject may reduce the prescribed dose, forget or stop treatment without informing the doctor, and without worrying about the short nor long-term consequences. This confirms the results of the literature, which reports that the side effects of treatment can be an obstacle to good therapeutic observance. According to Paquot, the occurrence of side effects is one of the main causes behind poor therapeutic observance (5). Shams et al. also confirm that the more subjects experience the occurrence of adverse effects, the more likely they are to abandon their treatment (29). This observation should also encourage health professionals, especially those treating patients, to adapt and simplify the treatment in accordance with the intricacies of the disease and the constraints declared by the diabetic subject, which prevent them from following their medication rigorously. Moreover, Scheen and Giet confirm that “the more effective and well-tolerated the treatment, the better it will be followed” (13). Thus, Palazzolo also insists on the importance of warning subjects with regards to the possibility of side effects to reduce their anxiety and at the same time improve their observance behavior (30).

It is shown that information on the disease, its complications, hygiene and dietary rules, medical treatment, and the consequences of non-observance of treatment is associated with therapeutic observance. Indeed, subjects who are not informed about their disease are more susceptible to abstain from following the recommendations related to medical treatment strictly. This observation leads us to a key point, the importance of therapeutic education, which must occupy health professionals and encourage them to improve the relationship between the practitioner and the diabetic subject and to think of targeted programs for therapeutic education. In the same context, the ENTRED study presents similar results, diabetic subjects who declared a need for information on the treatment of diabetes are twice as susceptible not to comply with their treatment (28).

The problem of access to medical care is a barrier to appropriate treatment. It is an important factor in relation to a failing health system. Respondents reported that the unavailability of medical treatments in the health care units, whether at the provincial hospital, health center, or local dispensary, due to stockouts, was the major cause of this problem. Thus, diabetic subjects who reported having this type of problem were three times more susceptible not to follow their medication correctly (OR=3 ; 95% CI [1.78–5.03]). In addition, these subjects reported that they did not have enough money to buy it at the pharmacy, so they were forced to have their dosage modified from the one prescribed by their doctor or stop it. This assessment gives us reason to approve, a priori, the efforts of the state and public health decision-makers to provide free access to certain drugs for chronic diseases, particularly diabetes and hypertension, especially in disadvantaged provinces like the one where this study was conducted. But also, to think seriously about how to remedy the problem of stock shortages, such as the use of a computerized system specialized in stock management in the different health care units of the province, which allows for a global vision of the stock level. It is also interesting to opt for a permanent inventory strategy.

Finally, we come to the understanding that environmental support is a factor that positively influences therapeutic observance. This finding is not surprising for a country such as Morocco, which is known for its social values, especially family support in case of chronic diseases (31). This result also shows the usefulness of improving and supporting therapeutic observance by integrating the entourage of the diabetic subject (32).

Improving therapeutic observance must be of greater concern to health professionals because non-observance can only lead to a worsening of the disease. Adapting and simplifying medical prescriptions in lights of the socioeconomic and psychosocial characteristics of the diabetic subject, based on the characteristics of the disease and according to the constraints declared by the subject and which prevent him/her from following the medical treatment rigorously, and a good strategy for therapeutic education can only have a positive impact on therapeutic observance.
